# Timing of Tracheostomy in Critically Ill Patients: A Meta-Analysis

**DOI:** 10.1371/journal.pone.0092981

**Published:** 2014-03-25

**Authors:** Huibin Huang, Ying Li, Felinda Ariani, Xiaoli Chen, Jiandong Lin

**Affiliations:** 1 Department of Critical Care Medicine, The First Affiliated Hospital of Fujian Medical University, Fuzhou, China; 2 Department of Pulmonary Medicine, Huadong Hospital, Shanghai Medical School of Fudan University, Shanghai, China; D’or Institute of Research and Education, Brazil

## Abstract

**Objective:**

To compare important outcomes between early tracheostomy (ET) and late tracheostomy (LT) or prolonged intubation (PI) for critically ill patients receiving long-term ventilation during their treatment.

**Method:**

We performed computerized searches for relevant articles on PubMed, EMBASE, and the Cochrane register of controlled trials (up to July 2013). We contacted international experts and manufacturers. We included in the study randomized controlled trials (RCTs) that compared ET (performed within 10 days after initiation of laryngeal intubation) and LT (after 10 days of laryngeal intubation) or PI in critically ill adult patients admitted to intensive care units (ICUs). Two investigators evaluated the articles; divergent opinions were resolved by consensus.

**Results:**

A meta-analysis was evaluated from nine randomized clinical trials with 2,072 participants. Compared to LT/PI, ET did not significantly reduce short-term mortality [relative risks (RR) = 0.91; 95% confidence intervals (CIs) = 0.81–1.03; p = 0.14] or long-term mortality (RR = 0.90; 95% CI = 0.76–1.08; p = 0.27). Additionally, ET was not associated with a markedly reduced length of ICU stay [weighted mean difference (WMD) = −4.41 days; 95% CI = −13.44–4.63 days; p = 0.34], ventilator-associated pneumonia (VAP) (RR = 0.88; 95% CI = 0.71–1.10; p = 0.27) or duration of mechanical ventilation (MV) (WMD = − 2.91 days; 95% CI = −7.21–1.40 days; p = 0.19).

**Conclusion:**

Among the patients requiring prolonged MV, ET showed no significant difference in clinical outcomes compared to that of the LT/PI group. But more rigorously designed and adequately powered RCTs are required to confirm it in future.

## Introduction

Tracheostomy is customarily performed when ICU patients require long-term ventilation and fail to remove the tracheal intubation in the near future. This procedure has become more widespread, because of the extensive application of percutaneous dilatational tracheostomy (PDT) performed at the patients’ bedside [1].

Tracheostomy is an invasive procedure that is associated with complications such as bleeding, infection, subcutaneous emphysema, pneumothorax and tracheal stenosis [2], [3]. However, compared to long-term translaryngeal intubation, tracheostomy may offer acknowledged advantages for critically ill patients, such as less airway dead space and lower airway resistance, thereby potentially reducing the work of breathing, decreasing analgesics and sedative requirements, avoiding oropharyngeal and laryngeal lesions, oral feeding possible, and providing easier and safer nursing care more comfortable to patients [4]–[6]. Furthermore, several studies demonstrated that early tracheostomy (ET) might shorten the duration of ventilation, and the length of ICU stay, and that ET might reduce the incidence of ventilation-associated pneumonia (VAP) and even mortality in critically ill patients [7]–[9]. However, these advantages remain controversial. Several studies have challenged the benefit of ET [10]–[14]. In these studies, ET was defined as tracheotomy performed from 48 hours to more than 3 weeks after the initiation of translaryngeal intubation. The difference regarding the timing of tracheostomy might lead to different outcomes. However, the potential benefit of optimal timing for performing tracheostomy in critically ill patients requiring ET or prolonged intubation (PI) has not been established.

Three meta-analyses have been published regarding the effect of the timing of tracheostomy on the prognosis of prolonged mechanically ventilated patients [15]–[17]. Of these studies, two studies [15], [16] defined ET as a tracheotomy conducted up to 7 days and one study [17] up to 10 days after the initiation of translaryngeal intubation; these studies assessed the influence of tracheostomy early or late on the incidence of mortality, the duration of mechanical ventilation (MV) and ICU stay and other important clinical outcomes in critically ill adult patients. However, inconclusive results were presented concerning several outcomes among the three meta-analyses. Recently, an increasing number of studies have been published concerning the timing of tracheotomy on the prognosis of critically ill patients. The majority of the patients were enrolled in RCTs. We, therefore, undertook an updated systematic review and meta-analysis of RCTs to determine whether tracheostomy performed at an earlier stage has significant benefits on important outcomes in critically ill patients.

## Methods

### Search Strategy, Inclusion and Exclusion Criteria

An extensive computer search of the literature was conducted, including PubMed, EMBASE and the Cochrane Library (up to July 2013). Manual searches of journals and reference lists were also performed. Authors of papers were contacted when the results were unclear or when relevant data were not reported. Searches were performed using multiple terms, including “tracheotomy” or “tracheostomy” and “ill patients” or “critical care” or “intensive care”. The search was limited to human subjects and RCTs. No language restriction was imposed. Finally, the websites of the international network were searched to ensure that all suitable trials were included.

Studies were eligible for inclusion in the present analysis if they met the following criteria: (1) research design: RCTs; (2) population: critically ill adult patients who were admitted to ICUs, and required prolonged MV; (3) intervention: patients were assigned to either an ET group or a late tracheostomy (LT)/PI group, regardless of the tracheotomy technique used such as surgical technique (ST) or PDT. We defined ET as a tracheostomy conducted within 10 days after the initiation of translaryngeal intubation, whereas LT was performed more than 10 days after the initiation of translaryngeal intubation; (4) studies should contain data for at least one of the following outcomes: mortality, duration of MV, length of ICU stay and VAP.

Studies were excluded for following reasons: (1) the studies were quasi-randomized clinical trials; (2) ET was performed more than 10 days after the initiation of translaryngeal intubation or LT was conducted within 10 days after the initiation of translaryngeal intubation; (3) the data were missing or incomplete or the study authors were unreachable or did not reply if additional information from their trials was required.

### Date Extraction, Quality and Risk-of-bias Assessment

Full-text versions of all eligible studies were obtained for quality assessment and the following data were independently extracted by two authors (HH and YL): first author, publication year, tracheostomy approach (PDT or ST), previously mentioned important clinical outcome data in our analysis, definition of VAP and methodological quality of the study. The extracted data were entered using Microsoft Excel 2010 and were checked by a third author (XC). Disagreement or doubt was resolved in pairs by consensus.

The methodological quality of the included studies was evaluated by two authors (HH and YL) using the Jadad 5-point scale, which consists of three items describing (1) randomization, (2) blinding, and (3) drop-outs and withdrawals in the report of a RCT. We assigned 2 points if the method of item was described and was appropriate; 1 point if the corresponding information of item was of insufficient detail and 0 point if the information was inappropriate. The quality scale ranged from 0 to 5 points. The studies were regarded to be of high quality if the Jadad score was ≥3 points and low quality if the score was ≤2 points [18].

The quality of studies was additionally examined using the method recommended by a Cochrane Collaboration tool for assessing risk of bias in the included RCTs. We assigned a value of ‘high’, ‘unclear’, or ‘low’ to the following items: (1) selection bias (Was there adequate generation of the randomization sequence?); (2) selection bias (Was allocation concealment satisfactory?); (3) performance and detection bias (Was there blinding of participants, personnel and outcome assessors?); (4) attrition bias (Were incomplete outcome data sufficiently assessed and dealt with?); (5) reporting bias (Was there evidence of selective outcome reporting?); and (6) other bias (Were any other sources of bias identified?) [19].

### Statistical Analysis

The primary outcomes were short-term mortality and long-term mortality, and the secondary outcomes included duration of MV, length of ICU stay (defined as the time from admission to discharge from the ICU) and VAP. To facilitate comparisons with the previous meta-analysis [16], we used the same definition of short-term mortality, and we referred to ICU or hospital mortality or mortality within a 90-day follow-up after admission. If a study reported all of these outcome measures, the longer observation period was preferred. Long-term mortality referred to mortality between hospital discharge and at least 1 year follow-up thereafter. Testing the robustness of our outcome and exploring the optimal timing of ET, we further assessed the effect of our outcomes by selecting studies with ET performed within 4 and 7 days.

The results from all of the relative studies were combined to estimate the relative risks (RRs) and associated 95% confidence intervals (CIs) for dichotomous outcomes such as incidence of mortality and VAP. With respect to the continuous outcomes of the duration of MV and ICU stay, weighted mean differences (WMDs) and 95% CI were estimated as the effect results. Heterogeneity was tested by using the I^2^ statistic, and studies were considered to have low (I^2^ = 25–49%), moderate (I^2^ = 50–74%) or high (I^2^≥75%) heterogeneity [20]. Thus an I^2^≥50% considered to indicate significant heterogeneity in a study, in which case a random-effect model was used, whereas an inverse variance method of fixed-effect model was used in cases where the outcome had no significant heterogeneity (I^2^<50%). Whenever heterogeneity was present, we performed sensitivity analyses to investigate the influence of a single study on the overall pooled estimate by excluding one study in each turn. Publication bias was assessed by funnel plot using mortality as an endpoint. P<0.05 was considered to be statistically significant in this meta-analysis. All of the statistical analyses were performed using STATA version 12.0 (STATA Corporation, College Station, TX, USA).

## Results

### Study Selection

**Figure 1:**
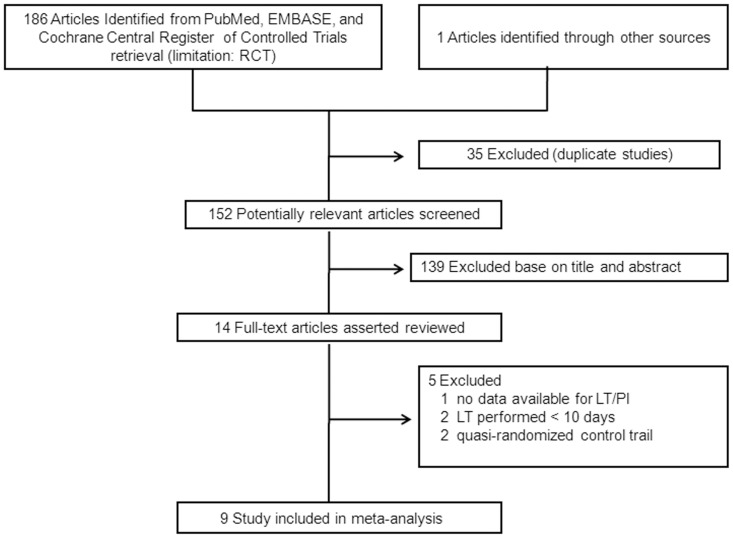
Selection process for randomized controlled trials included in the meta-analysis.

The initial search yielded 187 potentially relevant studies, of which 35 were excluded as duplicate studies and 139 were excluded based on the titles and abstracts. Thus, the full texts of fourteen studies were read for further evaluation, and five studies were excluded because two were quasi-randomized controlled trials [6], [21], two had ET and LT both performed within 10 days [22]–[23], and one had no data available for LT/PI. Consequently, nine RCTs [7]–[9], [11]–[13], [24]–[26] totaling 2,072 patients were included in our analysis. The flow chart of our search strategy is shown in Figure 1.

### Characteristics, Quality, and Bias Assessment of Included Studies

Table 1 presents the main characteristics of the nine RCTs finally included in this analysis. These studies enrolling a total of 2,072 patients (1,033 were in the ET group and 1,039 received LT/PI) were published between 2002 and 2013. The RCTs ranged in size from 44 patients to 909 patients. All of the nine studies were published in English. Five studies used PDT [7]–[9], [13], [25], two studies [11], [12] chose ST, and the remaining two studies [24], [25] chose either PDT or ST during their tracheotomy procedure. The studies in this meta-analysis enrolled various populations, including elective surgery patients [9], [13], nonselective critically ill medical patients [7], [11], [12], [25], trauma [8], [26] and burn [24] patients. All of the studies included reported the outcome of mortality; three studies [11]–[13] used mortality as a primary outcome measure. Different definitions of VAP were used among eight studies [7]–[9], [12], [13], [24]–[26]. The Centers for Disease Control and Prevention (CDC) criteria were adopted in three studies [8], [24], [26], whereas three studies [7], [12], [13] defined VAP based on clinical features with positive cultures of pulmonary secretion samples, and the remaining two studies [9], [25] used the simplified Clinical Pulmonary Infection Score (CPIS) to diagnose the presence of VAP if CPIS was >6.

**Table 1:** Summary Characteristics of the Study.

Study/yearpublished	Ref.No.	ICUsetting	Surgicalapproach	ET group	LT/PI group	Outcome pre-state/Jadad score	VAP definition
Young 2013	11	70 adult general and 2cardiothoracic CCU	PDT/ST	Within 4 days (n = 451)	After 10 days if still indicated(n = 448)	Mortality, length of ICU stay/3	Not reported
Zheng 2012	9	Surgical patients	PDT	Day 3 of MV (n = 58)	Day 15 of MV (n = 61)	Mortality, duration of MV, length ofICU stay, VAP/5	Using the modified CPIS.
Trouillet2011	13	Postcardiac surgeryICU	PDT	Before 5 days after surgery(n = 109)	15 d after initiation of MV(n = 107)	Mortality, duration of MV, length ofICU stay, VAP/4	Clinical features with positiveBAL cultures
Terragni 2010	25	12 ICUs	PDT	After 6–8 days of laryngealintubation (n = 209)	After 13–15 days of laryngealintubation (n = 210)	Mortality, duration of MV, length ofICU stay, VAP/4	Using the modified CPIS.
Bolt 2008	12	25 Medical or surgicalICUs	PDT/ST	Within 4 days (n = 61)	Prolonged endotrachealintubation (n = 62)	Mortality, duration of MV, length ofICU stay, VAP/3	Clinical features with positiveBAL cultures
Barquist 2006	26	Trauma center	ST	Before day 8 (n = 29)	After day 28 (n = 31)	Mortality, duration of MV, length ofICU stay, VAP/4	CDC criteria
Rumbak 2004	7	3 Medical ICUs	PDT	Within 48 hr (n = 60)	Days 14–16 of MV (n = 60)	Mortality, duration of MV, length ofICU stay, VAP/4	Clinical features with positiveBAL cultures
Bouderk2004	8	Units for head injurypatients	PDT	5–6 days after ICUadmission (n = 31)	Prolonged endotracheal intubation(n = 31)	Mortality, length of ICU stay/3	CDC criteria
Saffle 2002	24	Burn ICU.	ST	4 days after burn Injury(n = 21)	14 days after burn injury(n = 23)	Mortality, duration of MV, length ofICU stay, VAP/3	CDC criteria

ICU, intensive care unit; MV, mechanical ventilation; VAP, ventilator-associated pneumonia; CPIS, Clinical Pulmonary Infection Score; CDC, Centers for Disease Control and Prevention; ET, early tracheotomy; LT late tracheotomy; PI, prolonged intubation; PDT, percutaneous dilatational tracheostomy; ST, surgery technique; BAL, bronchoalveolar lavage.

**Figure 2:**
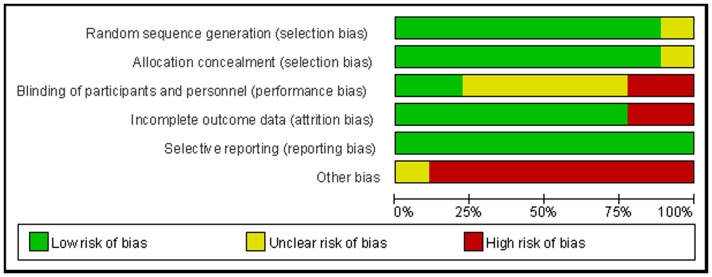
Risk of bias graph: the authors’ judgments about each risk-of-bias item presented as percentages across all included studies.

**Figure 3:**
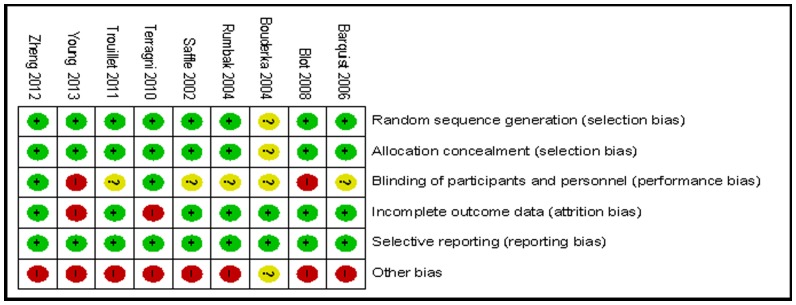
Risk-of-bias summary: the authors’ judgments about each risk-of-bias item for the included studies.

The Jadad scores of the studies (range, 3–5) are described in Table 1. Figure 2 and 3 show the overall methodological quality of the RCTs included by the Cochrane Collaboration tool for assessing risk of bias. All of the studies except one [8] described the methods of randomization and adequate allocation concealment, and only two studies [9], [25] were double-blinded. The risk of bias from selective reporting was small because the outcomes of interest in all of the included RCTs were adequately described.

### Primary Outcome: Mortality

**Figure 4:**
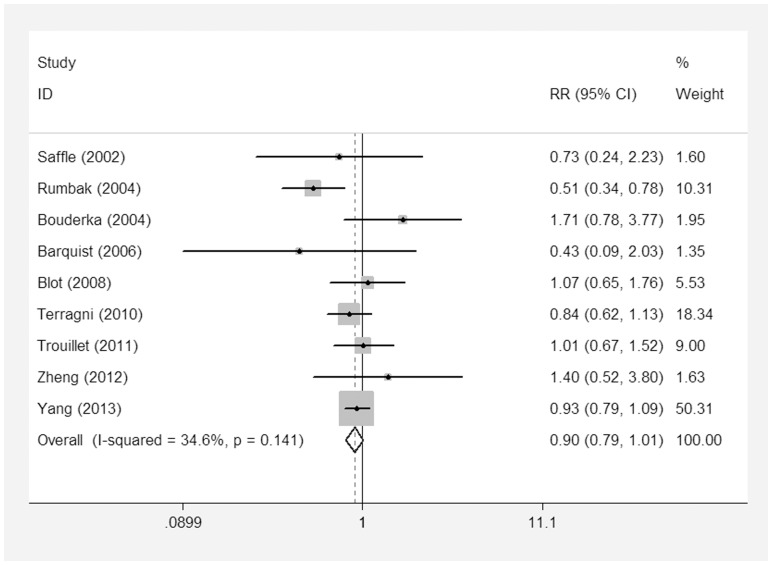
Forest plot showing the short-term mortality between the ET group and the LT/PI group.

**Figure 5:**
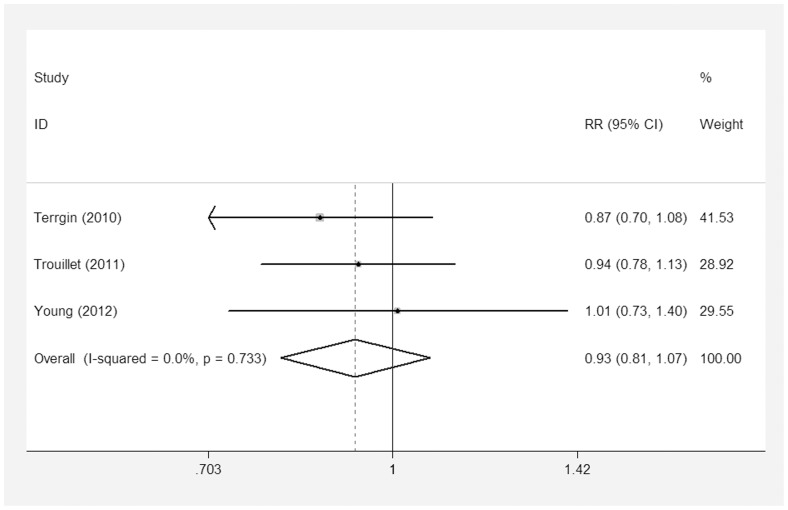
Forest plot showing the comparison of the long-term mortality between the ET group and the LT/PI group.

All of the nine studies [7]–[9], [11]–[13], [24]–[26] included 2,023 patients and reported short-term mortality. Of the 1,002 patients in the ET group, 322 died, compared to 359 of 1,021 patients who died in the LT/PI group. The pooled analysis suggested that ET did not reduce the short-term mortality (RR = 0.91; 95% CI = 0.81–1.03; p = 0.14) There was significant heterogeneity in this outcome (P for heterogeneity = 0.12; I^2^ = 34.6%) (Figure 4). Three of the pooled studies [11], [13], [25] had reported long-term outcomes of their patients (RR = 0.93; 95% CI = 0.81–1.07; p = 0.32, P for heterogeneity = 0.72; I^2^ = 0%) (Figure 5). There was no reduction in mortality when studies with ET performed within four days were obtained (RR = 0.84; 95% CI = 0.61–1.15; p = 0.28); these findings were similar to studies with ET performed within seven days (RR = 0.93; 95% CI = 0.83–1.04; p = 0.19) (Table 2).

**Table 2:** Effects of clinical outcomes between patients performed ET or LT/PI within 4, 7 or 10 days.

Subgroupanalysis	ET≤10 days andLT≥10 days			ET≤7 days andLT≥10 days			ET≤4 days andLT≥10 days		
	Trials,number	Patients,number	RR/WMD(95% CI), p	Trialsnumber	Patientsnumber	RR/WMD(95% CI), p	Trialsnumber	Patientsnumber	RR/WMD(95% CI), p
Short-termmortality	9	2,023	0.91 [0.81, 1.03], p = 0.14	7	1,544	0.93 [0.83, 1.04], p = 0.19	4	1,222	0.84 [0.61, 1.15], p = 0.28
Long-termmortality	3	994	0.93 [0.81, 1.07], p = 0.32	2	702	0.97 [0.81, 1.07], p = 0.79	1	551	–
Duration ofMV	6	621	−2.91 [−7.21, 1.40], p = 0.19	5	521	−3.57 [−8.28, 1.13], p = 0.14	2	239	−6.03 [−13.48, 1.42], p = 0.11
ICU stay	3	396	−4.41 [−13.44, 4.63], p = 0.34	2	336	−6.93 [−16.50, 2.63], p = 0.16	1	120	–
VAP	8	1,163	0.88 [0.71, 1.10], p = 0.27	6	684	0.87 [0.65, 1.15], p = 0.31	2	239	0.39 [0.13, 1.16], p = 0.09

ICU, intensive care unit; MV, mechanical ventilation; VAP, ventilator-associated pneumonia; ET, early tracheotomy; LT, late tracheotomy; PI, prolonged intubation; RR, relative risks; WMD, weighted mean difference.

### Secondary Outcomes: Duration of MV, ICU Stay and VAP

**Figure 6:**
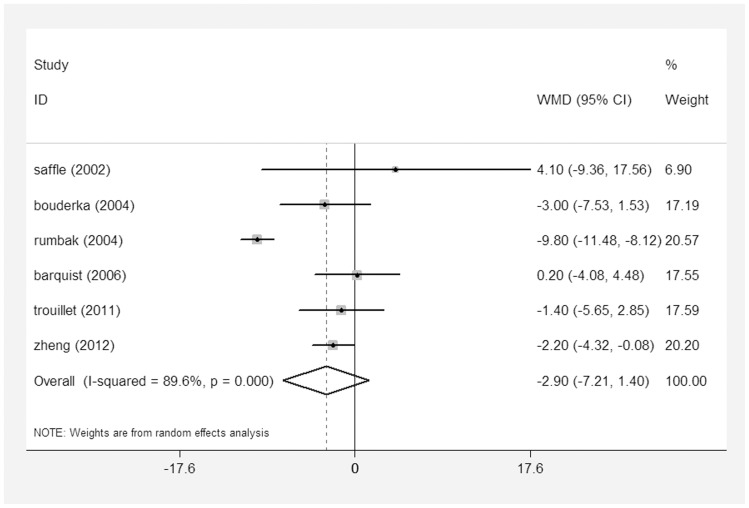
Forest plot showing the comparison of the duration of MV between the ET group and the LT/PI group.

Data that identified MV duration were available in the nine studies [7]–[9], [11]–[13], [24]–[26]; six studies reported mean (SD) time duration [7]–[9], [13], [24], [26], and three studies reported median (IQR) [11]–[12], [25]. Among the nine included trials, one trial had a mean MV duration of 1 week [7], whereas other studies [8], [9], [11]–[13], [24]–[26] had a mean MV duration of between 2 weeks and 5 weeks. No significant difference was detected between the ET group and the LT/PI group (WMD = −2.91 days; 95% CI = −7.21–1.40 days; p = 0.19). Significant heterogeneity in this outcome was observed among the included studies (I^2^ = 89.6%) (Figure 6).

**Figure 7:**
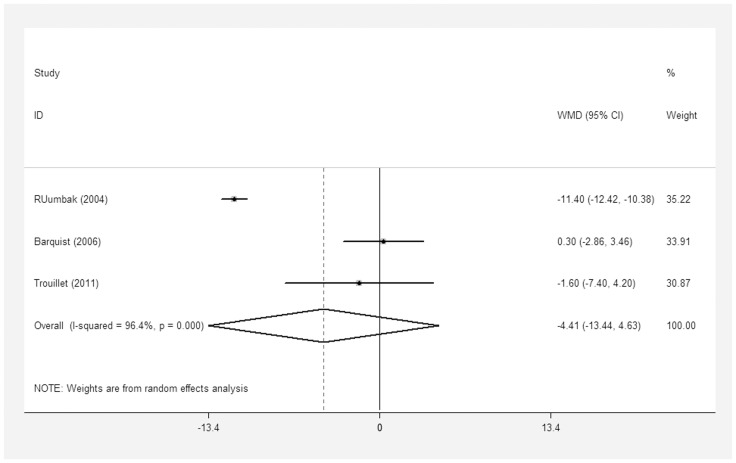
Forest plot showing the comparison of the length of ICU stay between the ET group and the LT/PI group.

Six studies included ICU stay as an outcome of interest [7], [9], [11], [13], [25]–[26], and three studies [9], [11], [25] reported median (IQR). Among the six included trials, the study by Rumbak et al. had a mean ICU stay of no more than 1 week [7], whereas other studies had a mean ICU stay of more than two weeks [9], [11], [13], [25], [26]. The aggregation of three studies [7], [13], [26] reported mean (SD) values showing that ET performed within 10 days was not associated with a significant reduction in ICU stay compared to the control group (WMD = −4.41 days; 95% CI = −13.44 to 4.63 days; p = 0.34), with significant heterogeneity among the studies (I^2^ = 96.4%) (Figure 7).

**Figure 8:**
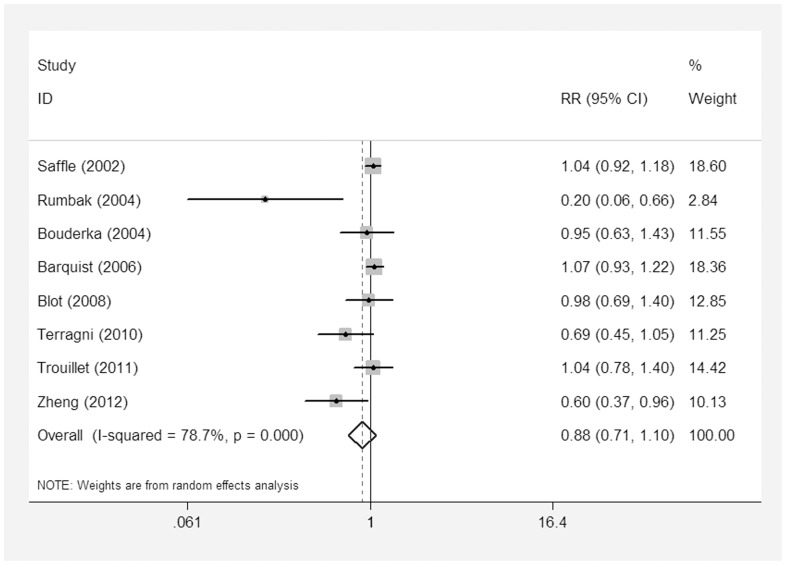
Forest plot showing the incidence of VAP between the ET group and the LT/PI group.

Eight studies [7]–[9], [12], [13], [24]–[26] evaluated the incidence of VAP. The incidence of VAP was not different in ET patients compared to those of the control group (RR = 0.88; 95% CI = 0.71–1.10; p = 0.27), with statistical evidence of heterogeneity among the studies (I^2^ = 78.7%) (Figure 8).

When only studies with ET performed within four or seven days were included to assess the effectiveness of the secondary outcomes, no significant differences were noted between the ET group and the control group (all p≥0.09) (Table 2).

### Sensitivity Analyses

**Figure 9:**
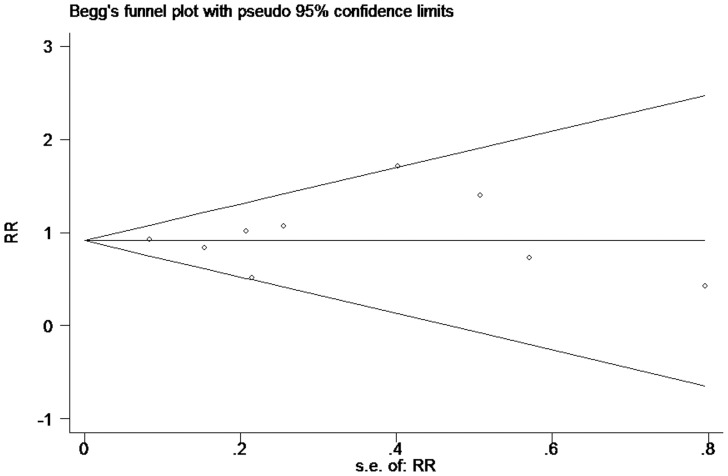
Tests for publication bias for RR of the shorter-term mortality.

We performed sensitivity analyses to explore the potential sources of heterogeneity. Exclusion of the study by Rumbak et al. [7] resolved the heterogeneity in short-term mortality, duration of MV and length of ICU stay (all P for heterogeneity >0.57; I^2^ = 0%). We found that results of short-term mortality and length of ICU stay had not been significantly changed except the duration of MV had shifted to −1.76 days (95% CI = −3.37 to −0.15 days; p = 0.03). Using mortality as an endpoint, the funnel plot did not suggest the presence of publication bias (Figure 9).

## Discussion

We investigated the influence of important clinical outcomes in critically ill adult patients who received an ET or LT during their treatment. Our meta-analysis showed that ET did not significantly reduce short-term or long-term mortality. Additionally, ET was not associated with a markedly reduced duration of MV, length of ICU stay and VAP.

Despite the acknowledged or controversial advantages, tracheostomy, either PDT or ST, had been extensively adopted for decades by many clinicians in their routine clinical practice. However, uncertainty exists with regard to the optimal timing and potential benefits of the tracheotomy in critically ill patients requiring prolonged ventilation. Thus, there is no consistency about specific timing of the tracheotomy either early or late. In fact, the timing of the tracheostomy varied. A survey conducted by Blot et al [27] in 152 French ICUs indicated that early tracheotomy (<3 weeks of MV) was considered by 68% of the respondents, after a median time of seven days. In a nationwide survey in 513 German ICUs, Kluge et al [28] found that the majority (68.2%) of tracheostomies were performed during the second week of MV and that 21.7% were performed by physicians during the first week. Additionally, tracheostomies were reportedly performed after a median period of 11 days in an international survey by Esteban et al. [29] and similarly in the Swiss study [30] (second week of MV) and in the study by Engoren and colleagues (median, 13–17 days) [31]. Currently, most physicians consider ET to be performed within 1–2 weeks after intubation; therefore, we defined ET as tracheotomy performed within 10 days after intubation in this meta-analysis.

To the best of our knowledge, three meta-analyses of ET performed in critically ill patients have been published [15]–[17]. However, interpretations of the results from the meta-analysis by Griffiths et al. [15] are limited because the study was based on only five trials with a total of 406 patients and two of these trials were quasi-RCTs [6], [20], thereby leading to a potential selection bias. The same apply to the meta-analysis by Gomes Sliva BN et al. [17] which pooled only four trials and one of these trials was quasi-RCTs [6]. In the two meta-analyses by Griffiths et al. [15] and Lu et al. [16], ETs were both defined as a tracheostomy conducted up to 7 days after the initiation of translaryngeal intubation, whereas ETs are currently considered by most physicians to be performed within 1–2 weeks after intubation [28]–[31]. However, in the meta-analysis by Lu et al. [16], the authors included a study [25] in which certain patients in the ET group had ET performed after 7 days; these patients represented 40.1% (419/1,044) of all enrolled patients in their analysis, thus decreasing the credibility of their results.

To provide a better characterization of the evidence base for ET for critically ill patients, we pre-stated rigorous inclusion criteria and included only RCTs that provided specific clinical outcomes. Therefore larger sample sizes were included, and nine RCTs comprising 2,072 participants were enrolled, thus giving greater statistical power to evaluate this effect.

Our meta-analysis indicated that tracheotomy performed at an early stage within 10 days did not significantly decrease the short-term mortality, which was consistent with previous meta-analyses. Exclusion of any single study (excluding each in turn) and sensitivity analyses based on various criteria did not significantly change the pooled results and may demonstrate sufficient robustness in our findings. Significant heterogeneity was observed among these studies. Our sensitivity analyses found that one study by Rumbak et al. likely contributed to this heterogeneity [7]. These authors claimed that several patients in their study were moved to a regular ward while they received MV; thus, the duration of ventilation was sometimes longer than the duration of ICU stay in the ET group. Differences in medical care between the ICU and the general ward might affect the prognosis of patients, which might lead to a selection bias between the ET group and the control group. Three pooled studies [11], [13], [25] had reported long-term mortality of patients. The limited evidence suggested that ET did not reduce long-term mortality. Therefore, more RCTs are required to explore the effect of ET on long-term prognosis.

The absence of a significant effect on the duration of MV and the length of ICU stay in this meta-analysis is contrary to the results of an earlier meta-analysis by Griffiths et al. [15], which observed a significant reduction of the duration of MV and the length of ICU stay. In their meta-analysis, only two trials provided data on the length of ICU stay and four trials provided data on the duration of MV in the ET group; both of these sets of trials included the same quasi-RCT [21]. Tentatively excluding the quasi-RCT in their meta-analysis would completely change the pooled results of the MV duration. The WMD of the MV duration shifted from −8.49 days (95% CI = −15.32 to −1.66; p = 0.03) to −4.94 days (95% CI = −11.64 to 1.77; p = 0.15).

However, our findings require additional consideration because significant heterogeneity was presented. In our sensitivity analyses, we noted that this heterogeneity was likely attributed to one trial conducted by Rumbak et al. [7] that was included in our meta-analysis. In their trial, several patients who transferred from the ICU were still receiving MV, thus leading to a shorter duration of MV and length of ICU stay than that observed in other studies. However, the fact that the duration of MV was significantly altered in the present analysis after the exclusion of that study [7] suggests insufficient robustness in our findings. Additionally, for tracheostomized patients, ICU discharge was not always determined by the condition of the patients. The hospital policy to accept or refuse tracheostomized patients in general wards and the availability of beds for patients requiring long-term tracheotomy or MV may affect the length of ICU stay [27]. These situations that are common in clinical practice, however, have not yet been reported in pooled studies.

Although advances have been made in the diagnosis, prevention and treatment strategies of VAP in recent years, VAP remains a serious problem in ICU patients, with reported incidence rates of 10% to 65% [7], [9], [12], [13], [32]–[33]. The influence of ET on VAP had been reported; however, these studies present inconclusive results. In our meta-analysis, we evaluated the efficacy of ET in preventing VAP in ICU patients requiring prolonged ventilation. We found that there was no significant difference in VAP rates between the ET and LT/PI group. Caution should be applied before interpreting the result as substantial heterogeneity was observed among these studies. This heterogeneity could be caused by differences in several aspects. First, different definitions of VAP were used among the eight studies [7]–[9], [12], [13], [24]–[26], which could lead to heterogeneity and subsequently influence the outcomes. Second, the patient populations varied across the included studies. Elective surgery patients, such as cardiac and other surgery requiring ICU admission postoperatively [9], [13], were usually in stable health condition; these types of patients might have enough time to make the necessary preparations, such as oral cavity cleaning, preoperative fasting and receiving oral antiseptic preoperatively compared to the nonselective ICU patients [7], [8], [12], [24]–[26]. Additionally, oral care scheme, timing of ventilator weaning, rescue sedative agents and the varying administration of antibiotics to treat infection in other sites might have contributed to this heterogeneity.

The strength of our meta-analysis is that we had defined rigorous inclusion criteria and incorporated original studies that enrolled large samples of patients in a randomized controlled design. We conducted additional analyses and noted the invalidity of ET performed in an earlier stage within 4 or 7 days. Nevertheless, several limitations of our meta-analysis should be considered. First, all of the RCTs included in our study were published in English; several trials [8], [19], [26] included a small number of patients (n<100). Second, there was considerable heterogeneity in our outcomes and insufficient robustness in several results, such as duration of MV. The targeted population was varied; the tracheotomy approach (PDT or ST) adopted and definitions of VAP were different across the studies. We had originally tried to perform subgroup analyses to explore studies according to such diversities. However, there were insufficient data. Third, only three studies [11]–[13] included in our meta-analysis used mortality as a primary outcome measure, and in the remaining six studies [7]–[9], [24]–[26], mortality was only one of the clinical endpoints. Moreover, only a small number of pooled studies offered relevant data in regard to clinical outcomes, such as length of ICU stay and long-term mortality, which weakened the statistical effect of these clinical outcomes.

Although our results showed no statistically significant difference, there is insufficient evidence to support or refute claims of clinical benefit of ET in critically ill patients due to significant heterogeneity and inconsistent definitions of the studies included. In addition, the questions that remain to be evaluated in more rigorously designed and adequately powered RCTs of ET performed in critically ill patients include the tracheotomy approach, type of patients, safety and complications.

### Conclusion

In summary, based on the available data, our meta-analysis suggested that ET as an intervention in critically ill adult patients did not reduce short-term or long-term mortality compared to LT/PI; moreover, incidence of VAP and duration of MV and ICU stay were also unaffected. Future RCTs are needed to define which subgroups of critically ill adult patients are most likely to benefit from this intervention.

## Supporting Information

Checklist S1 (DOC)

## References

[1] Al-Ansari MA, Hijazi MH (2006) Clinical review: Percutaneous dilatational tracheostomy. Crit Care 10: 202 10.1186/cc3900X 16356203 PMC1550816

[2] Terra RM, Fernandez A, Bammann RH, Castro AC, Ishy A, et al (2007) Open bedside tracheostomy: routine procedure for patients under prolonged mechanical ventilation. Clinics (Sao Paulo) 62 427–432: doi.org/10.1590/S1807–59322007000400009. 10.1590/s1807-59322007000400009 17823705

[3] Mallick A, Bodenham AR (2010) Tracheostomy in critically ill patients. Eur J Anaesthesiol 27: 676–82 10.1097/EJA.0b013e32833b1ba0 20523214

[4] MacIntyre NR, Cook DJ, Ely EW Jr, Epstein SK, Fink JB, et al (2001) Evidence-based guidelines for weaning and discontinuing ventilatory support: a collective task force facilitated by the American College of Chest Physicians; the American Association for Respiratory Care; and the American College of Critical Care Medicine. Chest 120: 375S–395S 10.1378/chest.120.6suppl.375S 11742959

[5] Nieszkowska A, Combes A, Luyt CE, Ksibi H, Trouillet JL, et al (2005) Impact of tracheotomy on sedative administration, sedation level, and comfort of mechanically ventilated intensive care unit patients. Crit Care Med 33: 2527–2533 10.1097/01.CCM.0000186898.58709.AA 16276177

[6] Dunham CM, La Monica C (1984) Prolonged tracheal intubation in the trauma patient. J Trauma 24: 120–124. 6694236 10.1097/00005373-198402000-00005

[7] Rumbak MJ, Newton M, Truncale T, Schwartz SW, Adams JW, et al (2004) A prospective, randomized, study comparing early percutaneous dilational tracheotomy to prolonged translaryngeal intubation (delayed tracheotomy) in critically ill medical patients. Crit Care Med 32: 1689–1694 10.1097/01.CCM.0000134835.05161.B6 15286545

[8] Bouderka MA, Fakhir B, Bouaggad A, Hmamouchi B, Hamoudi D, et al (2004) Early tracheostomy versus prolonged endotracheal intubation in severe head injury. J Trauma 57: 251–254 10.1097/01.TA.0000087646.68382.9A 15345969

[9] Zheng Y, Sui F, Chen XK, Zhang GC, Wang XW, et al (2012) Early versus late percutaneous dilational tracheostomy in critically ill patients anticipated requiring prolonged mechanical ventilation. Chin Med J (Engl) 125: 1925–1930 10.3760/cma.j.issn.03666999.2012.11.016 22884055

[10] Bösel J, Schiller P, Hook Y, Andes M, Neumann JO, et al (2013) Stroke-Related Early Tracheostomy Versus Prolonged Orotracheal Intubation in Neurocritical Care Trial (SETPOINT) A Randomized Pilot Trial. Stroke 44: 21–28 10.1161/STROKEAHA.112.669895 23204058

[11] Young D, Harrison DA, Cuthbertson BH, Rowan K; TracMan Collaborators (2013) Effect of early vs. late tracheostomy placement on survival in patients receiving mechanical ventilation: the TracMan randomized trial. JAMA 309: 2121–2129 10.1001/jama.2013.5154 23695482

[12] Blot F, Similowski T, Trouillet JL, Chardon P, Korach JM, et al (2008) Early tracheotomy versus prolonged endotracheal intubation in unselected severely ill ICU patients. Intensive Care Med 34: 1779–1787 10.1007/s00134-008-1195-4 18592210

[13] Trouillet JL, Luyt CE, Guiguet M, Ouattara A, Vaissier E, et al (2011) Early percutaneous tracheotomy versus prolonged intubation of mechanically ventilated patients after cardiac surgery: a randomized trial. Ann Intern Med 154: 373–383 10.7326/0003-4819-154-6-201103150-00002 21403073

[14] Plummer AL, Gracey DR (1989) Consensus conference on artificial airways in patients receiving mechanical ventilation. Chest 96: 178–180 10.1378/chest.96.1.178 2500308

[15] Griffiths J, Barber VS, Morgan L, Young JD (2005) Systematic review and meta-analysis of studies of the timing of tracheostomy in adult patients undergoing artificial ventilation. BMJ 330: 1243 10.1136/bmj.38467.485671.E0 15901643 PMC558092

[16] Wang F, Wu Y, Bo L, Lou J, Zhu J, et al (2011) The timing of tracheotomy in critically ill patients undergoing mechanical ventilation: a systematic review and meta-analysis of randomized controlled trials. Chest 140: 1456–1465 10.1378/chest.112024 21940770

[17] Gomes Silva BN, Andriolo RB, Saconato H, Atallah AN, Valente O (2012) Early versus late tracheostomy for critically ill patients. Cochrane Database Syst Rev. 2012 Mar 14 3: CD007271 10.1002/14651858.CD007271 22419322

[18] Kjaergard LL, Villumsen J, Gluud C (2001) Reported methodologic quality and discrepancies between large and small randomized trials in meta-analyses. Ann Intern Med 135: 982–989 10.7326/0003-4819-135-11-200112040-00010 11730399

[19] Higgins JP, Altman DG, Gøtzsche PC, Jüni P, Moher D, et al (2011) Cochrane Bias Methods Group; Cochrane Statistical Methods Group et al (2011) The Cochrane Collaboration’s tool for assessing risk of bias in randomized trials. BMJ 343: d5928 10.1136/bmj.d5928 22008217 PMC3196245

[20] Higgins JP, Thompson SG, Deeks JJ, Altman DG (2003) Measuring inconsistency in meta-analyses. BMJ 327: 557–560 10.1136/bmj.327.7414.557 12958120 PMC192859

[21] Rodriguez JL, Steinberg SM, Luchetti FA, Gibbons KJ, Taheri PA, et al (1990) Early tracheostomy for primary airway management in the surgical critical care setting. Surgery 108: 655–659. 2218876

[22] Bösel J, Schiller P, Hook Y, Andes M, Neumann JO, et al (2013) Stroke-related early tracheostomy versus prolonged orotracheal Intubation in neurocritical care trial (SETPOINT): a randomized pilot trial. Stroke 44: 21–28 10.1161/STROKEAHA 23204058

[23] Koch T, Hecker B, Hecker A, Brenck F, Preuβ M, et al (2012) Early tracheostomy decreases ventilation time but has no impact on mortality of intensive care patients: a randomized study. Langenbecks Arch Surg 397: 1001–1008 Doi: 10.1007/s00423-011-0873-9. 22322214 10.1007/s00423-011-0873-9

[24] Saffle JR, Morris SE, Edelman L (2002) Early tracheostomy does not improve outcome in burn patients. J Burn Care Rehabil 23: 431–438 10.1097/01.BCR.0000036586.83628.F9 12432320

[25] Terragni PP, Antonelli M, Fumagalli R, Faggiano C, Berardino M, et al.. (2010) Early vs late tracheotomy for prevention of pneumonia in mechanically ventilated adult ICU patients: a randomized controlled trial. doi: 10.1001/jama.2010.447.JAMA 303: 1483–1489. 10.1001/jama.2010.447 20407057

[26] Barquist ES, Amortegui J, Hallal A, Giannotti G, Whinney R, et al (2006) Tracheostomy in ventilator dependent trauma patients: a prospective, randomized intention-to-treat study. J Trauma 60: 91–97 10.1097/01.ta.0000196743.37261.3f 16456441

[27] Blot F (2005) Melot C; Commission d’Epidémiologie et de Recherche Clinique (2005) Indications, timing, and techniques of tracheostomy in 152 French ICUs. Chest 127: 1347–1352 10.1007/s00134-008-1195-4 15821214

[28] Kluge S, Baumann HJ, Maier C, Klose H, Meyer A, et al (2008) Tracheostomy in the intensive care unit:a nationwide survey. Anesth Analg 107: 1639–1643 10.1213/ane.0b013e318188b818 18931225

[29] Esteban A, Anzueto A, Alía I, Gordo F, Apezteguía C, et al (2000) How is mechanical ventilation employed in the intensive care unit? an international utilization review. Am J Respir Crit Care Med 161: 1450–1458 10.1164/ajrccm.161.5.9902018 10806138

[30] Fischler L, Erhart S, Kleger GR, Frutiger A (2000) Prevalence of tracheostomy in ICU patients: a nation-wide survey in Switzerland. Intensive Care Med 26: 1428–1433 10.1007/s001340000634 11126252

[31] Keenan SP, Powers C, McCormack DG, Block G (2002) Noninvasive positive-pressure ventilation for postextubation respiratory distress: a randomized controlled trial. JAMA 287: 3238–3244 10.1001/jama.287.24.3238 12076220

[32] Chastre J, Fagon JY (2002) Ventilator-associated pneumonia. Am J Respir Crit Care Med 165: 867–903 10.1164/ajrccm.165.7.2105078 11934711

[33] Safdar N, Dezfulian C, Collard HR, Saint S (2005) Clinical and economic consequences of ventilator-associated pneumonia: A systematic review. Crit Care Med 33: 2184–2193 10.1097/01.CCM.0000181731.53912.D9 16215368

