# Supplementary material for: Timing of Tracheostomy in Critically Ill Patients: A Meta-Analysis
Source: PLoS One. 2014 Mar 25;9(3):e92981. doi: 10.1371/journal.pone.0092981 (PMC3965497; doi:10.1371/journal.pone.0092981)
Supplement: Checklist S1 — (DOC) [file pone.0092981.s001.doc]

| **Section/topic** | **#** | **Checklist item** | **Reported on page #** |
| --- | --- | --- | --- |
| **TITLE** | | |  |
| Title | 1 | Timing of tracheotomy in critically ill patients: A Meta-analysis of Randomized Controlled Studies | Title |
| **ABSTRACT** | | |  |
| Structured summary | 2 | *Context:* Potential benefit of optimal timing for performing tracheotomy in critically ill patients still remains unclear.  Objective: To compare the effectiveness of clinical important outcomes between early tracheotomy (ET) and late tracheotomy (LT) or prolonged intubation (PI) for critically ill patients receiving long-term ventilation during their treatment.  *Data Sources:* An extensive computer search of the literature was conducted, including PUBMED, EMBASE and the Cochrane Library (up to July 2013). Manual searches of journals and reference lists were also performed. Authors of papers were contacted when results were unclear or when relevant data were not reported. Searches terms included tracheotomy, tracheotomy and ill Patients, critical care or intensive care.  Study Selection: Studies eligible for inclusion if they met the following criteria: (1) research design: RCTs; (2) population: critically ill adult patients ad­mitted to ICU, require prolonged MV; (3) intervention: patients were assigned to either ET group or LT/PI group, regardless of the tracheotomy technique such as surgery technique (ST) or PDT. We defined ET as a tracheotomy conducted within 10 days after initiation of translaryngeal intubation, while LT was more than 10 days; (4) included studies should at least contain one of the following outcome data (mortality, duration of MV, length of ICU stay and VAP).  *Data Extraction:* Independent extraction of articles by 2 authors using predefined data fields, including study quality indicators.  *Data Synthesis:* A meta-analysis was evaluated from nine randomized clinical trials with 2,072 participants. Compared with LT/PI, ET did not significantly reduce short-term mortality (RR 0.91; 95% CI 0.81, 1.03; p = 0.14) or long-term mortality (RR 0.90; 95% CI 0.76, 1.08; p = 0.27). In addition, ET was not associated with a markedly reduced length of ICU stay (WMD -4.41 days, 95% CI -13.44 to 4.63 days, P = 0.34), ventilator-associated pneumonia (VAP) (RR, 0.88; 95% CI, 0.71 to 1.10; P = 0.27) and duration of mechanical ventilation (MV) (WMD -2.91 days, 95% CI -7.21 to 1.40 days, P = 0.19) were also observed.  *Conclusion:* Among patients requiring prolonged MV, ET showed no significant difference in clinical important outcomes comparing with that of LT/PI group. | Abstract |
| **INTRODUCTION** | | |  |
| Rationale | 3 | “Compared with a long time of translaryngeal intubation, some studies also found that ET may shorten duration of ventilation, length of ICU stay, and reduce incidence of ventilation-associated pneumonia (VAP) and even mortality in critically ill patients. However, these advantages still remains controversial. There were studies that challenged the controversial benefit of ET. From these studies, ET was defined as tracheotomy performed ranging from 48 hours to more than three weeks after initiation of translaryngeal intubation. The difference regarding the timing of tracheotomy might lead to different outcomes. However, potential benefit of optimal timing for performing tracheotomy in critically ill patients requiring ET or PI still remains unclear.” | Introduction |
| Objectives | 4 | “…We undertook an update systematic review and meta-analysis of RCTs to determine whether tracheotomy performed at an earlier stage has significant benefits on the clinical important outcomes in critically ill patients.” | Introduction |
| **METHODS** | | |  |
| Protocol and registration | 5 | N/A | N/A |
| Eligibility criteria | 6 | *Types of studies:* “The search was limited to human subjects and RCTs. No language restriction was imposed.”  *Types of participants:* “critically ill adult patients ad­mitted to ICU, require prolonged MV”  *Types of intervention:* “patients were assigned to either ET group or LT/PI group, regardless of the tracheotomy technique such as surgery technique (ST) or PDT. We defined ET as a tracheotomy conducted within 10 days after initiation of translaryngeal intubation, while LT was more than 10 days.”  *Types of outcome measures:* “Primary outcome measures: mortality；Secondly outcome measures: duration of MV, length of ICU stay and VAP.” | METHODS  Search Strategy, inclusion and exclusion criteria |
| Information sources | 7 | “Studies were identified by searching electronic databases, scanning reference lists of articles and consultation with experts in the field….. No limits were applied for language. This search was applied to PUBMED, EMBASE and the Cochrane Library (up to July 2013). The last search was run in July 2013. In addition, the websites of the international network were searched to ensure that all suitable trials were included.” | METHODS  Search Strategy, inclusion and exclusion criteria |
| Search | 8 | *“*We used the following search terms to search all trials registers and databases: tracheotomy, tracheotomy and ill Patients, critical care or intensive care…’’  The flow chart of search strategy is shown in Figure 1 | METHODS  Search Strategy, inclusion and exclusion criteria |
| Study selection | 9 | “Full-text versions of all eligible studies were obtained for quality assessment and data extraction was independently performed by two authors…, Disagreement or doubt was resolved in pairs by consensus.” | METHODS  Search Strategy, inclusion and exclusion criteria |
| Data collection process | 10 | “Full-text versions of all eligible studies were obtained for quality assessment and data extraction was independently performed by two authors…, Disagreements between reviewers were resolved.” by consensus. Extracted data were entered into Microsoft Excel 2010 and were checked by a third author. Disagreement or doubt was resolved in pairs by consensus.” | METHODS  Date Extraction, Quality and risk-of-bias |
| Data items | 11 | “Information was extracted from each included trial on: “first author, publication year, tracheotomy approach (PDT or ST), pre-stated important clinical outcomes data in our analysis, definition of VAP and methodological quality of the study.” | METHODS  Date Extraction, Quality and risk-of-bias assessment |
| Risk of bias in individual studies | 12 | “Methodological quality of the included studies was evaluated by Huibin Huang and Ying Li using the Jadad 5 point scale, which consists of three items describing (1) randomization; (2) blinding, and (3) drop-outs and withdrawals in the report of a RCT…”  “The quality of studies was additionally examined with the method recommended by a Cochrane Collaboration tool for assessing risk of bias in included RCTs....” | METHODS  Date Extraction, Quality and risk-of-bias |
| Summary measures | 13 | “The results from all the relative studies were combined to estimate the relative risks (RRs) and associated 95% confidence intervals (CIs) for dichotomous outcomes such as incidence of mortality and VAP. With respect to the continuous outcomes of duration of MV and ICU stay, weighted mean differences (WMDs) and 95% CI were estimated as the effect results.” | METHODS  Statistical Analysis |
| Synthesis of results | 14 | “Heterogeneity was tested by using the I2 statistic, and studies were considered to low (I^2^=25-49%), moderate (I^2^=50-74%) and high (I^2^≥75%) heterogeneity. Thus an I^2^≥50% was regarded as indicating significant heterogeneity in this study and a random-effect model was used while an inverse variance method of fixed-effect model was used in case when the outcome had no significant heterogeneity (I^2^ <50%).” | METHODS  Statistical Analysis |

| **Section/topic** | **#** | **Checklist item** | **Reported on page #** |
| --- | --- | --- | --- |
| Risk of bias across studies | 15 | “Publication bias was assessed by funnel plot using mortality as an endpoint.” | METHODS  Statistical Analysis |
| Additional analyses | 16 | “Testing the robustness of our outcome and explore the optimal timing of ET, we further assessed the effect of our outcomes by choosing studies with ET performed within four and seven days….Whenever heterogeneity was present, we carried out sensitivity analyses to investigate the influence of a single study on the overall pooled estimate by excluding of one study in each turn.” | METHODS  Statistical Analysis |
| **RESULTS** | | |  |
| Study selection | 17 | “The initial search yielded 187 potentially relevant studies, of which 35 were excluded for duplicate studies and 139 studies were excluded based on the titles and abstracts. Thus, fourteen studies were full-text read for further evaluation, and five of them were excluded because two were quasi-randomized control trails, two because ET and LT were performed both within 10 days, one had no data available for LT/PI. Consequently, nine RCTs, totaling 2,072 patients were included in our analysis. The flow chart of search strategy is shown in (Fig1).” | Results—Study Selection, fig 1 |
| Study characteristics | 18 | “Table 1 summarizes the main characteristics of the nine RCTs finally included in this analysis. These studies enrolling a total of 2,072 patients (1,033 to ET group and 1,039 receiving LT/PI) were published between 2002 and 2013. The sizes of the RCTs ranged from 44 to 909 patients. All the nine studies were published in English. PDT was used in five studies, two studies chose ST and the remaining two studies chose either PDT or ST during their tracheotomy procedure. The studies in this meta-analysis enrolled various populations, including elective surgery patients, nonselective critical ill medical patients, trauma and burn patients. All the studies included reported the outcome of mortality, among which three used mortality as a primary outcome measure. Different definitions of VAP were used among eight studies. The Centers for Disease Control and prevention (CDC) criteria were adopted in three studies, whereas three studies defined VAP based on clinical features with positive cultures of pulmonary secretion samples and the remaining two studies used the simplified Clinical Pulmonary Infection Score (CPIS) to diagnose the presence of VAP if CPIS > 6.” | Results—Characteristics, quality, and bias assessment of Included Studies |
| Risk of bias within studies | 19 | See Figure 2-3. | Figure2-3 |
| Results of individual studies | 20 | See Table 1-2 and Figure 4-9. | Table1-2  Figure4-9 |
| Synthesis of results | 21 | “Primary outcome:  All the nine studies included 2,023 patients reported the short-term mortality. Of the 1,002 patients in the ET group, 322 died, compared to 359 out of 1,021 patients in the LT/PI group. The pooled analysis suggested that ET did not reduce the short-term mortality (RR 0.91; 95% CI 0.81, 1.03; p = 0.14) There was significant heterogeneity for this outcome (P for heterogeneity = 0.12; I^2^ = 34.6%) (Fig4). Three of the pooled studies had reported the long-term outcomes of their patients (RR 0.93; 95% CI 0.81, 1.07; p = 0.32, P for heterogeneity=0.72; I^2^ = 0%).  Secondary outcomes:  Duration of MV, ICU stay and VAP: Data identified duration of MV were available in all the nine studies, of which six reported mean (SD) time duration, and three reported median (IQR). Among the nine included trials, one had a mean duration of MV about 1 week, whereas other studies had mean duration of MV between 2 and 5 weeks. No significant difference was detected between the ET group and LT/PI group. (WMD -2.91 days, 95% CI -7.21 to 1.40 days, P = 0.19). Significant heterogeneity was observed in this outcome among the included studies (I^2^= 89.6%). Six studies included ICU stay as an outcome of interest, three of them reported median (IQR). Among the six included trials, the study by Rumbak et al. had a mean length of ICU stay no more than 1 week, whereas other studies had mean length of ICU stay more than two weeks. Aggregation of three studies reported mean (SD) showed that ET performed within 10 days was not associated with significant reduction of ICU stay compared with control group (WMD -4.41 days, 95% CI -13.44 to 4.63 days, P = 0.34), with significant heterogeneity among the studies (I^2^=96.4%).Eight studies evaluated the incidence of VAP. The incidence of VAP was not different in ET patients compared with those of control group (RR, 0.88; 95% CI, 0.71 to 1.10; P = 0.27), with statistical evidence of heterogeneity among the studies (I^2^ = 78.7%).” | Results- Primary outcome: Mortality  - Secondary outcomes: Duration of MV, ICU stay and VAP |
| Risk of bias across studies | 22 | “Using mortality as an end point, the funnel plot did not suggest the presence of publication bias (Fig9).” | Results- Sensitivity analyses，Figure9 |
| Additional analysis | 23 | “There was no reduction in mortality when studies with ET performed within four days were obtained (RR 0.84; 95% CI 0.61, 1.15; p = 0.28), these findings were similar in studies with ET performed within seven days (RR 0.93; 95% CI 0.83, 1.04; p = 0.19)….. When only studies with ET performed within four or seven days were included to assess the effectiveness of the secondary outcomes, no significant differences were found between ET group and control group (all p values≥0.09)…. We performed sensitivity analyses to explore potential sources of heterogeneity. Exclusion of study by Rumbak et al. resolved the heterogeneity in short-term mortality, duration of MV and length of ICU stay (all P for heterogeneity >0.57; I^2^ = 0%). In the mean while, we found that results of short-term mortality and length of ICU stay had not been significantly changed except duration of MV shifted to -1.76 days (95% CI -3.37 to-0.15 days, P=0.03).” | Results，Table 2 |
| **DISCUSSION** | | |  |
| Summary of evidence | 24 | “We investigated the influence of clinical important outcomes in critically ill adult patients who received an early or late tracheotomy during their treatment. Our meta-analysis found that early tracheotomy did not significantly reduce short-term or long-term mortality. In addition, ET was not associated with a markedly reduced duration of MV, length of ICU stay and VAP.” | Discussion |
| Limitations | 25 | “Several limitations of our meta-analysis should be taken into account. First, our analysis is based on RCTs that only published in English and some of them have included the relatively small number of patients (n <100). Second, there was considerable heterogeneity in our outcomes and insufficient robustness to some of our results such as duration of MV. The targeted population varied greatly, tracheotomy approach (PDT or ST) adopted and definitions of VAP were different across the studies. We had originally tried to perform sub­group analyses to explore studies according to such diversities. However, there were insufficient data. Third, only three studies included in our meta-analysis have used mortality as a primary outcome measure, and to the remaining six studies, mortality is just one of the clinical end points. Moreover, only a small number of pooled studies offered relevant data in regard to clinical outcomes such as length of ICU stay and long-term mortality, which weakened statistical effect of these clinical outcomes. Therefore, more RCTs are necessary to reveal the effect.” | Discussion |
| Conclusions | 26 | “In summary, based on the available data, our meta-analysis suggested that the ET did not reduce short-term or long-term mortality as an intervention towards critically ill adult patients compared with the LT/PI, incidence of VAP, duration of MV and ICU stay. Future RCTs are needed to define which subgroups of critically ill adult patients who are most likely to benefit from this intervention.” | Discussion- Conclusion |
| **FUNDING** | | |  |
| Funding | 27 | No current funding sources for this study. | NA |
